# Lipidomic Profiling of Red Blood Cells in the Mitochondrial Fatty Acid β‑oxidation Disorder MCADD Reveals Phospholipid and Sphingolipid Dysregulation

**DOI:** 10.1021/acs.jproteome.5c00308

**Published:** 2025-07-24

**Authors:** Inês M. S. Guerra, Helena B. Ferreira, Luísa Diogo, Sónia Moreira, Stefano Bonciarelli, Laura Goracci, Tânia Melo, Pedro Domingues, M. Rosário Domingues, Ana S. P. Moreira

**Affiliations:** a Mass Spectrometry Center, LAQV-REQUIMTE, Department of Chemistry, 56062 University of Aveiro, Campus Universitário de Santiago, Aveiro 3810-193, Portugal; b CESAM - Centre for Environmental and Marine Studies, Department of Chemistry, 56062 University of Aveiro, Campus Universitário de Santiago, Aveiro 3810-193, Portugal; c Centro de Referência de Doenças Hereditárias do Metabolismo, Unidade Local de Saúde de Coimbra, MetabERN, Coimbra 3000-075,Portugal; d Serviço de Bioquímica, Faculdade de Medicina da Universidade de Coimbra, Coimbra 3004-531,Portugal; e Molecular Discovery Ltd., Hertfordshire WD6 4PJ, U.K.; f Department of Chemistry, Biology and Biotechnology, 9309 University of Perugia, Perugia 06123, Italy

**Keywords:** lipids, lipidomics, mass spectrometry, FAOD, medium-chain acyl-CoA dehydrogenase deficiency, erythrocytes, glycerophospholipids, sphingolipids, plasmanyl, plasmenyl

## Abstract

Medium-chain acyl-CoA dehydrogenase deficiency (MCADD) is characterized by the accumulation of medium-chain acylcarnitines. Despite the therapeutic approach, changes in lipid homeostasis have been reported in MCADD plasma samples. Compared to plasma lipidomics, red blood cell (RBC) profiling provides a more stable biomarker that is less influenced by dietary changes and reflects long-term metabolic alterations. In this study, we assessed the plasticity of the lipidomic profile of RBC from children with MCADD and controls using C18 liquid chromatography–mass spectrometry. The results revealed significant alterations in 240 lipid species in MCADD, highlighting an upregulation of sphingolipids (sphingomyelins and ceramides) and lysophospholipid species (lysophosphatidylcholines and lysophosphatidylethanolamines) alongside a downregulation of polyunsaturated and ether-linked phosphatidylcholines (PCs) and phosphatidylethanolamines (PEs). Also, altered PC/PE and (PC + SM)/(PE + PS) ratios could be associated with alterations in RBC membranes properties, e.g., fluidity and asymmetry. The observed changes in the lipidome suggest compromised antioxidant defenses, enhanced oxidative stress, and an inflammatory state, with potential systemic implications in MCADD lipid metabolism and long-term complications in older age. This study underscores the utility of RBC lipidomics as a robust tool for understanding the pathophysiology of MCADD. It may prove to be a useful tool for monitoring disease progression in the near future.

## Graphical abstract

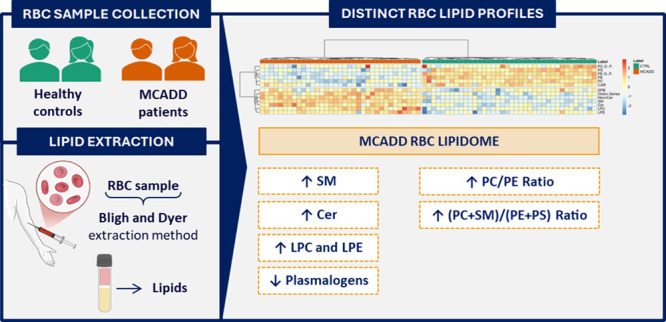

## 1 Introduction

Fatty acid β-oxidation disorders (FAODs) are a group of rare inherited metabolic diseases caused by defects in mitochondrial fatty acid β-oxidation. Medium-chain acyl-CoA dehydrogenase deficiency (MCADD) is the most common FAOD.^1–3^ This enzymatic deficiency results in a reduction of an important alternative energy production, during periods of glucose depletion, accompanied by elevated concentrations of specific medium-chain acylcarnitines (CARs) and fatty acids (FAs).^4,5^ The spectrum of clinical manifestations is wide. Most MCADD patients are asymptomatic, but severe clinical phenotypes can present within the first 2 years of life, usually during an acute illness.^6^ Clinical manifestations include severe hypoglycemia, vomiting, hypoglycemia-associated seizures, lethargy, and coma.^1^ Acute noninflammatory encephalopathy with hyperammonemia, liver dysfunction, and fatty infiltration of the liver may occur in patients with hepatomegaly and acute liver failure. Symptomatic patients with MCADD can also develop muscular hypotonia, supraventricular arrhythmias, and sudden death.^1,7^ Therapeutic approach consists of avoidance of fasting with frequent feeding and carbohydrate supplementation in periods of undercurrent illness.^1^ Long-term comorbidities, such as obesity and intellectual deficiency, can have a negative impact on the quality of life. Therefore, the disclosure of prognostic biomarkers is of utmost importance to improve the prognosis.

The MCADD involves disrupted lipid metabolism, marked by accumulation of specific CAR and FA.^1,4,8^ As FAs are components of complex lipids, changes in their profile may contribute to the pathology of MCADD. Indeed, alterations in lipid profile of MCADD patients have been documented in various biological samples, including blood plasma and dried blood spots (DBSs),^9–13^ in a trend similar to the one reported for other FAODs (LCHADD, VLCADD, and CTP2D).^14,15^ However, the long-term effects of the elevated CAR and FA levels in MCADD remain unclear. Few studies have suggested that these lipids may accumulate in the rat cerebral cortex, concomitant with decreased nonenzymatic antioxidant defenses, linked to enhanced oxidative stress,^8,16–20^ possibly leading to consequences at brain/cognitive level. Also, the accumulation of FA may lead to their esterification into complex lipids, causing alteration of the complex lipid profile.

Recent lipidomics and metabolomics studies have shown MCADD-related lipid metabolism alterations in several lipid classes, including glycerophospholipids, sphingolipids, and glycerolipids,^11–13^ . Notably, MCADD patients exhibit reduced plasma levels of sphingomyelin (SM) and ether-linked lipids, particularly phosphatidylcholine (PC) and phosphatidylethanolamine (PE) plasmalogens, which have important functions as endogenous antioxidants.^21^ Furthermore, significant increases in plasmatic PC and triglyceride (TG) species bearing saturated and monounsaturated FAs were also reported in MCADD patients.^13^ Moreover, elevated levels of oxidized PC have also been detected in DBSs of MCADD newborns.^11^ These lipid alterations can disrupt the characteristic lipidome of cells and organelles, contributing to cellular dysfunction and potential long-term clinical consequences, such as cardiovascular diseases, obesity, and type II diabetes mellitus.

Plasma samples are nowadays the preferred sample type for revealing the overall metabolic changes. However, red blood cells (RBCs) have increasingly been used to detect disease-related adaptations in the lipidome, offering notable advantages over plasma in studying of metabolic disorders.^22,23^ Their extended lifespan, approximately 120 days, provides a more robust and stable representation of the metabolic status, being less influenced by short-term dietary variations.^24,25^ RBCs have a simple cellular structural organization, consisting of a two-dimensional membrane with a cytoskeleton and phospholipid bilayer, distinguished by the complete absence of cellular organelles.^26,27^ Due to the lacking ability of synthesizing lipids,^28^ RBC lipid composition predominantly reflects the systemic lipid metabolism. As RBC exchange lipids with other cells,^26,29^ the RBC membrane lipids serve as a source of potential noninvasive biomarkers for monitoring long-term lipid metabolisms in other tissues.^30^ In fact, studies have shown that RBC lipidome can mirror the lipid changes occurring in tissues and other cell types, including neurons, glial cells,^26^ and retinal and optic nerves.^29^ Also, the lipid composition together with the membrane asymmetry of RBCs plays a critical role in preserving cell shape and functionality. These characteristics are known to be disrupted in a variety of pathological conditions, such as type 2 diabetes and coronary heart diseases.^31–33^ Additionally, lipid changes in RBC membranes have been reported in various disorders, including sickle cell disease, leukemia, heart failure, type 2 diabetes, and nonalcoholic fatty liver disease,^30,34–37^ and were also associated with the risk of developing cardiovascular disease.^35,38,39^ Thus, a detailed analysis of the of RBC lipidome is not only important for better understanding the membrane biology of these cells, but also for evaluating the pathophysiological alterations and disease prognosis.^26,40^

As MCADD is associated with alterations of the lipid profile, this study aims to assess the plasticity of the RBC lipidome in children with MCADD and identify potential prognostic biomarkers. For this purpose, the RBC lipidome from MCADD patients was compared with those from control children (CTRL) using the advanced lipidomic approaches based on the C18-liquid chromatography-high resolution mass spectrometry approach.

## 2 Material and Methods

### 2.1 Reagents

HPLC-grade acetonitrile (CH_3_CN, ACN), dichloromethane (CH_2_C_l2_), methanol (CH4O, MeOH), and isopropanol (C_3_H_8_O, IPA) were purchased from Fisher Scientific (Leicestershire, UK). Milli-Q water was obtained using the Milli-Q Millipore system (Synergy, Millipore Corporation, Billerica, MA, USA). Ammonium molybdate ((NH_4_)­6MoO_4_·4H_2_O) was purchased from Panreac (Madrid, Spain), and ascorbic acid (C_6_H_8_O_6_) was purchased from VWR Chemicals (Leuven, Belgium). Perchloric acid (HClO_4_, 70%) was purchased from Fisher Scientific (Leicestershire, UK). Sodium dihydrogen phosphate dihydrate (NaH_2_PO_4_·2H_2_O) was purchased from Riedel-de Han (Seelze, Germany). Ammonium formate (CH_5_NO_2_) and formic acid (CH_2_O_2_) were purchased from Sigma-Aldrich (St. Louis, MO, USA). Phospholipid internal standards 1,2-dimyristoyl-*sn*-glycero-3-phosphocholine (dMPC, PC 14:0/14:0), 1-nonadecanoyl-2-hydroxy-*sn*-glycero-3-phosphocholine (LPC 19:0), *N*-heptadecanoyl-D-*erythro*-sphingosylphosphorylcholine (SM 18:1;O2/17:0), 1,2-dimyristoyl-*sn*-glycero-3-phosphoethanolamine (dMPE, PE 14:0/14:0), 1,2-dimyristoyl-*sn*-glycero-3-phosphate (dMPA, PA 14:0/14:0), 1,2-dipalmitoyl-*sn*-glycero-3-phosphatidylinositol (dPPI, PI 16:0/16:0), 1,2-dimyristoyl-*sn*-glycero-3-phospho-(10-rac)­glycerol (dMPG, PG 14:0/14:0), 1,2-dimyristoyl-*sn*-glycero-3-phospho-l-serine (dMPS, PS 14:0/14:0), *N*-heptadecanoyl-D-*erythro*-sphingosine (Cer 18:1;O2/17:0), and 10,30-bis­[1–dimyristoyl-*sn*-glycero-3-phospho]-glycerol (tMCL, (CL14:0)_4_) were purchased from Avanti Polar Lipids, Inc. (Alabaster, AL, USA).

### 2.2 Red blood cell (RBC) samples

Red blood cell (RBC) samples were collected from 73 participants, comprising 35 individuals diagnosed with MCADD treated at the Reference Center for Hereditary Metabolic Diseases in Coimbra, Portugal, and 38 controls. The MCADD group included children aged between 4 months and 18 years, with a mean age of 7.65 years. This cohort consisted of 18 females and 17 males. The majority of patients (71.4%) were of Gypsy ethnicity and shared homozygosity for the c.985A > G (p.K329E) pathogenic variant. All 35 MCADD patients were on a regular diet and received carbohydrate supplementation (oral maltodextrins, raw cornstarch, or intravenous glucose) during episodes of intercurrent illness or other conditions that put them at risk for hypoglycemia due to glycogen depletion. Twenty-three patients did not require carbohydrate supplementation within 6 months prior to blood sampling, while 12 had intercurrent illnesses requiring oral carbohydrate supplementation in the same time period (7 within the past month). One patient required hospitalization for intravenous glucose 1.5 months prior to sample collection. Most patients had a normal body mass index, although two were overweight and two were obese. All but seven patients received L-carnitine supplementation, with doses ranging from 250 to 1500 mg/day, based on plasma monitoring conducted once or twice a year. Blood samples were obtained under normal dietary conditions during routine outpatient visits when patients were in a clinically stable state. Additional demographic and clinical patient data are provided in Supplementary Table S1.

The control group consisted of 38 healthy individuals, aged 3 months to 17 years (mean age 9.27 years), with 11 females and 27 males. These individuals underwent blood collection for unrelated reasons (e.g., minor surgical procedures), were in good health, were not taking any medications, and were following a normal diet. Blood samples were collected after fasting, which ranged from 1 to 14 h (mean 5.4 h, median 4 h) in MCADD patients and 2 to 12 h (mean 4.5 h, median 4 h) in controls. Two milliliters of blood was drawn into EDTA tubes, centrifuged at 2000g for 10 min to separate RBCs, and stored at −80 °C for subsequent lipid profile analysis.

The collection of RBC samples required for this study was accepted by the Hospital Ethics Committee (vote number: OBS.SF.023/2023).

### 2.3 RBC Total Lipid Extraction

Total lipids were extracted using a modified Bligh and Dyer method.^41^ Red blood cells (RBCs, 200 μL) were washed with ice-cold milli-Q water and centrifuged (14,000g, 10 min, 4 °C) (B. Braun Biotech International GmbH, Berlin, Germany). The washing procedure was repeated three times to obtain a clear supernatant. The pellet was recovered and resuspended in ice-cold Milli-Q water (1 mL). Lipid extraction was then initiated by adding ice-cold CH_2_Cl_2_:MeOH (1:2, v/v, 3.75 mL) to the sample, followed by incubation on ice (30 min) under orbital shaking (75 rpm) with periodic vortexing. Phase separation was achieved by sequential addition of CH_2_Cl_2_ (1.25 mL) and Milli-Q water (1.25 mL), followed by centrifugation (540 g, 10 min). The lower organic phase was collected, and a second extraction of the aqueous phase was performed with ice-cold CH_2_Cl_2_ (1.88 mL). The combined organic phases were dried under a N_2_ stream after removal of residual aqueous droplets by centrifugation. The extract was resuspended in CH_2_Cl_2_, transferred to an amber glass vial, dried under N_2_, and stored at −20 °C until analysis.

### 2.4 Phospholipid Quantification by Phosphorus Measurement

Total phospholipid (PL) quantification was performed through phosphorus measurement using a modified Bartlett and Lewis method.^42^ The total lipid extracts were dissolved in CH_2_Cl_2_ (200 μL), and aliquots (10 μL) were transferred in duplicate to glass tubes previously decontaminated with a nitric acid solution (5%). After solvent evaporation under nitrogen stream, perchloric acid (70%, 125 μL) was added to each tube, and samples were digested in a heating block (Stuart, U.K.) at 180 °C for 1 h. After cooling to room temperature, the following reagents were sequentially added with vortex mixing between each addition: Milli-Q water (825 μL), ammonium molybdate solution (2.5% w/v in Milli-Q water, 125 μL), and ascorbic acid solution (10% w/v in Milli-Q water, 125 μL). The samples were then incubated in a water bath at 100 °C for 10 min, followed by immediate cooling in a cold-water bath. The absorbance was measured at 797 nm using a Multiskan GO1.00.38 Microplate Spectrophotometer (Thermo Scientific, Hudson, NH, USA) controlled by SkanIT software, version 3.2 (Thermo Scientific). The phosphorus content of each extract was determined using a calibration curve prepared with phosphorus standards (0.1 to 2 μg of P) from a sodium dihydrogen phosphate dihydrate stock solution (100 μg mL^–1^ of P). The total phospholipid content was estimated by multiplying the phosphorus amount by 25.^43^

### 2.5 Characterization of the Total Lipidome Profile by Reverse-Phase Liquid Chromatography-High-Resolution Tandem Mass Spectrometry (C18 LC-MS/MS)

The total lipid extracts from RBC samples were analyzed using reverse-phase liquid chromatography coupled with high-resolution tandem mass spectrometry (C18-LC-MS/MS). An Ultimate 3000 Dionex system (Thermo Fisher Scientific, Bremen, Germany) equipped with an Ascentis Express C18 column (90 Å, 2.1 × 100 mm, 2.7 μm; Sigma-Aldrich) was paired with a Q-Exactive hybrid quadrupole Orbitrap mass spectrometer (Thermo Fisher Scientific, Bremen, Germany) for this purpose. Lipid extracts were resuspended in CH_2_Cl_2_to achieve a phospholipid concentration of 1 μg PL μL^–1^, and a 5 μL aliquot of a prepared mixture was injected for analysis. The mixture consisted of 10 μL of lipid extract (1 μg μL^–1^), 82 μL of isopropanol/methanol (1:1, v/v), and 8 μL of a phospholipid standard mix containing specific lipid standards at defined concentrations (1,2-dimyristoyl-*sn*-glycero-3-phosphocholine, 0.04 μg; *N*-heptadecanoyl-D-*erythro*-sphingosylphosphorylcholine, 0.04 μg; 1,2-dimyristoyl-snglycero-3-phosphoethanolamine, 0.04 μg; 1-nonadecanoyl-2-hydroxy-*sn*-glycero-3phosphocholine, 0.04 μg; 1,2-dipalmitoyl-*sn*-glycero-3-phosphoinositol, 0.08 μg; 1′,3′-bis­[1,2-dimyristoyl-*sn*-glycero-3-phospho]-glycerol, 0.16 μg; 1,2-dimyristoyl-*sn*-glycero-3-phosphoglycerol, 0.024 μg; *N*-heptadecanoyl-D-*erythro*-sphingosine, 0.08 μg; 1,2-dimyristoyl-*sn*-glycero-3-phospho-l-serine, 0.08 μg; 1,2-dimyristoyl-*sn*-glycero3-phosphate, 0.16 μg).

The separation was performed at 50 °C at a flow rate of 260 μL/min. The mobile phase A was Milli-Q water/acetonitrile (40:60%) with 10 mM ammonium formate and 0.1% formic acid, and mobile phase B was isopropanol:acetonitrile (90:10%) with the same additives. The gradient started at 32% B and increased to 45% B at 1.5 min, 52% B at 4 min, 58% B at 5 min, 66% B at 8 min, 70% B at 11 min, 85% B at 14 min, and 97% B at 18 min, which was maintained until 25 min. The system returned to 32% B at 25.01 min and underwent an 8 min re-equilibration before the next injection.

Mass spectrometry was performed in both positive (3.0 kV electrospray voltage) and negative (2.7 kV electrospray voltage) ionization modes. The capillary temperature was set to 320 °C, the probe temperature to 300 °C, and gas flows were set at 35 and 3 U for sheath and auxiliary gases, respectively. The S-lens RF level was 50 U. Full-scan data were acquired at a resolution of 70,000, with an *m*/*z* range of 200–1600, an automatic gain control (AGC) target of 3 × 10^6^, a maximum injection time (IT) of 100 ms, and 2 micro scans. Tandem mass spectrometry (MS/MS) spectra were obtained at a resolution of 17,500 with an AGC target of 1 × 10^5^, 1 micro scan, and the same maximum IT. Data were acquired in a cycle of one full scan followed by 10 data-dependent MS/MS scans, with dynamic exclusion set to 30 s and an intensity threshold of 8 × 10^4^. The normalized collision energy ranged from 20 to 28 eV in the negative mode and from 25 to 30 eV in the positive mode.

Data acquisition was performed using the Xcalibur data system (V3.3, Thermo Fisher 6 Scientific, Bremen, Germany). The LC-MS data were identified by using the Lipostar2 software (Molecular Discovery Ltd., version 2.1.5). The software was used to process raw data, detect peaks, align chemical features, identify lipid species, and measure peak areas. Lipid identification was carried out in both positive and negative ionization modes by comparing raw MS/MS data files to a reference spectral library generated with the DB Manager module of Lipostar by importing the LIPID MAPS structure database (accessed on May 14, 2024) that was further fragmented accordingly to the fragmentation rules encoded in the Lipostar software. The raw data were imported and aligned with the following parameters: MS signal filtering thresholds of 10,000 for positive mode and 5000 for negative mode; MS/MS signal filtering threshold of 100. Peaks were smoothed using the Savitzky-Golay method (window size: 7, degree: 2, and multipass iterations: 1) and detected with an *m*/*z* tolerance of 5 ppm. Isotope clustering was performed with a 10 ppm tolerance and a retention time (RT) tolerance of 0.2 min. Sample alignment settings included a 10 ppm tolerance and an RT tolerance of 0.5 min. The MS/MS filter was employed to selectively preserve features with MS/MS spectra for identification, with precursor ion and product ion mass tolerances set to 5 and 10 ppm, respectively. Lipid identifications were rated with a quality score of 3 to 4 stars. Finally, peak areas from the extracted ion chromatograms (XIC) were exported and normalization was performed using the area of a selected lipid internal standard.

### 2.6 Statistical Analysis

Multivariate and univariate statistical analyses were performed using R version 4.3.1 ^44^ in RStudio version 2024.04.2.^45^ The areas of lipid species (excluding neutral lipids such as TG, DG, and CE) were first normalized to selected lipid internal standards, scaled by the total area sum, then log_1__0_-transformed^46^ and subsequently normalized using EigenMS.^47^ Lipid class ratio data were similarly normalized to the total area sum, log_1__0_-transformed, and adjusted using EigenMS. The R packages FactoMineR^48^ and factoextra^49^ were used to perform principal component analysis (PCA). Heatmaps were created from autoscaled data using the R package pheatmap,^50^ using “Euclidean” as the clustering distance and “ward.D” as the clustering method. Data normality and variance homogeneity were assessed using Shapiro-Wilk and Levene’s tests, respectively. Based on these tests, data were analyzed using the Welch *t* test if assumptions were met or the Mann–Whitney test if not. *P* values were corrected for multiple testing using the Benjamin–Hochberg method for the false discovery rate (FDR, *q*-values). All univariate analyses were performed using the r package rstatix^51^ with a significance threshold of *p* < 0.05. All graphics were created using the R package ggplot2.^52^

#### 2.6.1 BioPAN Analysis of Lipidomic Data

Lipid pathway analysis was performed using the open access web-based tool, Bioinformatics Methodology For Pathway Analysis (BioPAN).^53^ The quantitative LC-MS/MS data were loaded into the BioPAN platform, on LIPID MAPS Lipidomic Gateway (https://lipidmaps.org/biopan/, accessed on 24 January 2025). The sphingoid base of SM lipid species was loaded considering the previously reported for RBC lipidome, with saturated SM containing the sphingoid base d18:0 and unsaturated SM containing d18:1.^54–56^ The following options were selected: MCADD as a condition of interest; CTRL as a control condition, lipid type, active status, subclass level, and reaction subset of lipid data; *p* value 0.05; and no paired data.

## 3 Results

### 3.1 Polar Lipidome Profile of Red Blood Cells (RBCs) from MCADD Patients and Control (CTRL) Individuals Using C18 LC-MS/MS

The lipidomics analysis of the RBC of MCADD and control (CTRL) children was done by high-resolution C18-RP-LC-MS and MS/MS. A total of 267 lipid molecular species (*m*/*z* values) were identified, distributed across 8 lipid classes and 21 lipid subclasses, including fatty acyl carnitines (CARs); phosphatidylcholine (PC), comprising diacyl PC, lyso-PC (LPC), alkyl-acyl (indicated with the prefix “O-” or also named as plasmanyl), and alkenyl-acyl species (indicated with prefix “P-” or also named as plasmenyl); phosphatidylethanolamine (PE), including diacyl-PE, lyso-PE (LPE), alkyl-acyl. and alkenyl-acyl species; phosphatidylserine (PS); sphingoid bases (SPB), comprising sphinganines, sphingosines. and sphingoid base homologues and variants; sphingomyelin (SM); ceramide (Cer); hexosylceramide (HexCer); dihexosylceramide (Hex_2_Cer); and globo series, comprising globo-triaosylceramide (Gb3) and globo-tetraosylceramides (Gb4). For most of the identified lipid species, the fatty acyl composition was determined using MS/MS data, as shown in Supplementary Table S2. The lipid species were semiquantified, and the statistical analysis was performed to assess the discrimination between MCADD and CTRL groups.

The unsupervised principal component analysis (PCA) (Figure 1) revealed a clear separation between the MCADD and CTRL groups. The MCADD group was plotted in the negative values of dimension 1 (Dim 1), while the CTRL appeared in the positive values of Dim 1. In the PCA score plot, the first dimension (Dim 1) accounted 62.4% of variance and the second dimension (Dim 2) account for 4.3% of variance in the overall study population, providing evidence of the lipidome plasticity of MCADD RBC. According to the loading values, significant contributors to Dim 1 were PC 40:7, PE P-40:7, PE 40:7, PC O-40:7/PC P-40:6, PC 40:8, PE 40:5, PC O-44:5/PC P-44:4, LPC 16:1, PC O-38:7/PC P-38:6, CAR 20:2, PE 36:4_A, PC 38:5_B, PC O-38:5/PC P-38:4, Gb4 38:1;O2, PC 24:0, and PC 36:6 (Supplementary Figure S1), while the main contributors to Dim 2 were CAR 12:0, SM 30:0;O2, PC 40:7, Hex2Cer 42:2;O2_B, PS 40:5, CAR 20:2, Gb4 40:1;O2, SM 30:1;O2, Gb4 36:1;O2, CAR 14:0, PS 38:5, PE P-40:7, LPE 22:4, PC O-38:7/PC P-38:6, Cer 40:0;O2, and SM 32:0;O2 (Supplementary Figure S2).

**Figure 1:**
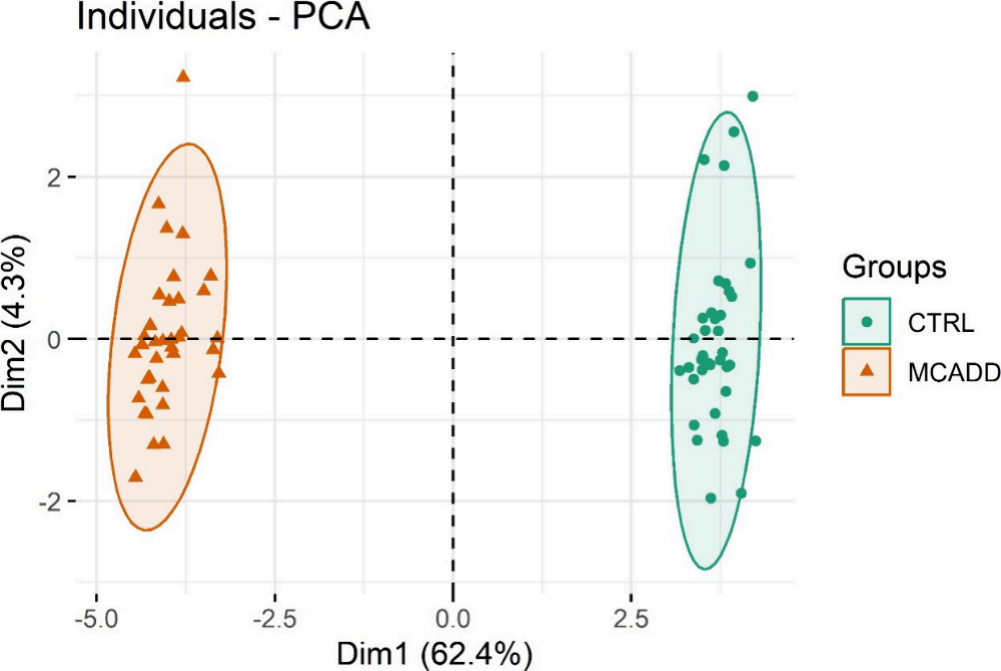
Principal component (PCA) score plot of the lipid species data set obtained using C18 RP-LC-MS/MS analysis of total lipid extracts from red blood cell samples of control (CTRL, green) and medium-chain acyl-CoA dehydrogenase deficiency (MCADD, orange) children.

Univariate analysis showed that 240 out of 267 identified lipid species varied significantly with a *q*-value <0.05 (Supplementary Table S3). The Welsh *t* test results were analyzed to identify the top 50 lipid species with the lowest *q*-values (*q* < 0.001). These 50 selected lipid species and most discriminant of the conditions were used to generate a two-dimensional hierarchical clustering heatmap (Figure 2). These 50 lipid species comprised 40 glycerophospholipids (14 PC, 9 ether-linked PC, 1 LPC, 8 PE, 7 ether-linked PE, and 1 LPE) and 10 sphingolipids (7 SM, 2 Cer, and 1 HexCer). The samples were independently clustered into two groups at the top of the hierarchical dendrogram, with the CTRL group (green) on the left and the MCADD groups (orange) on the right (Figure 2). The dendrogram on the left side of the heatmap illustrates the clustering of lipid species based on the similarity of the variations. The first upper cluster included mostly lipid species belonging to sphingolipid classes that were upregulated in MCADD RBC. The upregulated sphingolipid species included seven SM (SM 39:1;O2, SM 36:1;O2, SM 40:1;O2, SM 38:1;O2, SM 38:0;O2, SM 34:0;O2 and SM 34:1;O2), two Cer (Cer 39:1;O2 and Cer 38:1;O2), and HexCer 34:1;O2. Also, one short-chain saturated PC lipid species (PC 24:0) and two lysophospholipids (LPC 16:1 and LPE 18:1) were upregulated in MCADD RBC.

**Figure 2:**
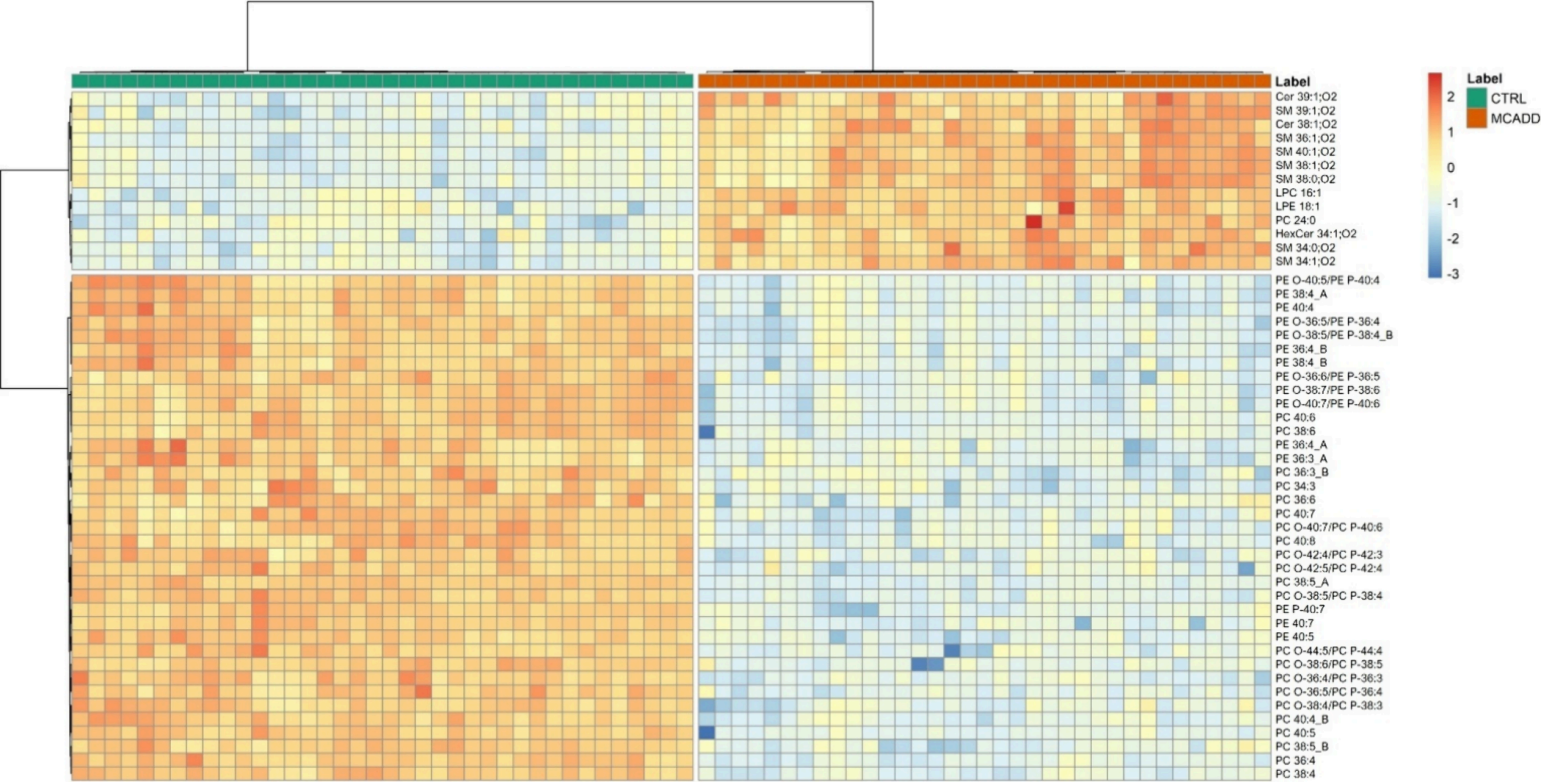
Two-dimensional hierarchical clustering heatmap of the 50 most discriminative (*q*-values <0.001) lipid species between the control (CTRL, in green) and medium-chain acyl-CoA dehydrogenase deficiency (MCADD, in orange) children. The relative abundance levels are depicted on the red-yellow-blue scale, with numbers indicating the fold difference from the overall mean. The red color indicates high abundance, while blue indicates low abundance. Yellow represents null values. The clustering of the control and disease groups is represented by the dendrogram at the top, while the clustering of individual lipid species is represented by the dendrogram on the left. The lipid species are labeled as follows: AAAA xx:i (AAAA = lipid class abbreviation; xx = number of total carbon atoms in FA; i = number of total double bonds). The prefix O- is used for plasmanyl species to indicate the presence of an alkyl ether substituent, whereas the prefix P- is used for plasmenyl species to indicate the alk-1-enyl ether substituent. The suffix “A” or “B” is used to differentiate isomers with distinct elution times. The abbreviations of lipid classes are as follows: Cer, ceramides; HexCer, hexosylceramide; LPC, lysophosphatidylcholine; LPE, lysophosphatidylethanolamine; PC, phosphatidylcholine; PE, phosphatidylethanolamine; and SM, sphingomyelin. The fatty acyl chain composition of each lipid species can be found in Supplementary Table S2.

The second cluster revealed a downregulation of 37 lipid species, all belonging to the phospholipid class (including PC and PE). Within the PC class, 23 lipid species, comprising 13 diacyl PC and 9 ether-linked PC, showed downregulation in MCADD patients. The PE class also exhibited a downregulation in the MCADD group compared to the CTRL group, with eight diacyl-PE and seven ether-linked PE altered.

Indeed, a trend for a variation at the level of lipid classes was observed with analysis of lipid species data set. To assess the plasticity of lipid classes in MCADD, a two-dimensional hierarchical clustering analysis was done considering 13 lipid subclasses out of the initial 21 (Figure 3). The ether-linked LPC and LPE were excluded due to an insufficient number of lipid species. Additionally, for SPB, we decided to group all species into a single subclass. The heatmap showed a clear separation between the MCADD and CTRL groups, highlighting the lipidome plasticity in MCADD at lipid subclass level. MCADD showed a downregulation of RBC phospholipid subclass PC, ether-linked PC, PE, and ether-linked PE and PS and a decrease in CAR, consistent with the trends observed in the lipid species heatmap (Figure 2). In contrast, a significant upregulation of several sphingolipid subclasses, including SPB, SM, Cer, HexCer, and globo series lipids, was observed in MCADD compared to CTRL. In addition to the increase in sphingolipid classes, an increase in lysophospholipid classes LPC and LPE was also observed in the RBC of MCADD patients, when compared to CTRL individuals.

**Figure 3:**
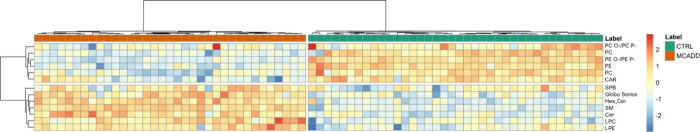
Two-dimensional hierarchical clustering heatmap of the 13 lipid subclasses showing variation between the control (CTRL, in green) and medium-chain acyl-CoA dehydrogenase deficiency (MCADD, in orange) children. The relative abundance levels are depicted on the red–yellow–blue scale, with numbers indicating the fold difference from the overall mean. The red color indicates high abundance, while blue indicates low abundance. Yellow represents null values. The clustering of the CTRL and MCADD groups is represented by the dendrogram at the top, while the clustering of individual lipid subclasses is represented by the dendrogram on the left. The abbreviations of lipid subclasses are as follows: CAR, acylcarnitines; Cer, ceramides; Globo series, including globo-triaosylceramide (Gb3) and globo-tetraosylceramides (Gb4); Hex_*n*_Cer, including hexosylceramide and dihexosylceramide; LPC, lysophosphatidylcholine; LPE, lyso phosphatidylethanolamine; PC, phosphatidylcholine; PE, phosphatidylethanolamine; PC O-/PC P-, ether-linked phosphatidylcholine; PE O-/PE P-, ether-linked phosphatidylethanolamine; PS, phosphatidylserine; and SM, sphingomyelin.

Considering that an increase in LPC lipid subclass, formed by degradation of PC species, is generally associated with increase of phospholipase A2 activity that can cause a decrease in PC lipid subclass levels,^57^ the PC/LPC ratio was calculated using log-transformed data. When comparing the ratios of the two groups, we observed that MCADD patients show a decrease in the PC/LPC ratio (*p* values <0.0001, Figure 4A).

**Figure 4:**
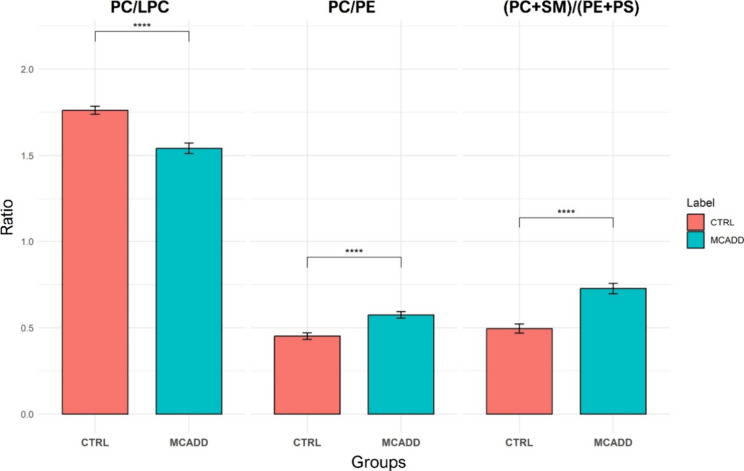
Phosphatidylcholine (PC) to lysophosphatidylcholine (LPC) ratio, phosphatidylcholine (PC) to phosphatidylethanolamine (PE) ratio, and phosphatidylcholine (PC) plus sphingomyelin (SM) to phosphatidylethanolamine (PE) to phosphatidylserine (PS) ratio of lipid extracts from red blood cell samples of control (CTRL) and medium-chain acyl-CoA dehydrogenase deficiency (MCADD) children. Ratios were calculated by using log10 and EigenMS transformed data. Significant differences between the groups are indicated by horizontal lines and denoted as **** if *q*-values <0.0001.

Certain lipid class ratios, such as PC/PE and (PC + SM)/(PE + PS), have been shown to be characteristic of RBC membrane phenotyping and to play a crucial role in keeping membrane properties like asymmetry and fluidity.^31,58^ In particular, cells can modify their membrane lipids to regulate the fluidity by adjusting the balance between ordered-crystalline-phase lipids (PE) and liquid-crystalline-phase lipids (PC).^59^ Thus, the PC/PE ratio was calculated and found to be significantly higher in MCADD patients (*q*-value <0.0001) compared to CTRL (Figure 4B). To further investigate whether RBC membrane asymmetry is affected in MCADD, the (PC + SM)/(PE + PS) ratio was compared between the two groups (Figure 4C). The results revealed significant differences (*q* value <0.001), with MCADD patients exhibiting a higher ratio than the CTRL group.

BioPAN calculates statistical scores for all possible lipid pathways to predict which are active or suppressed in MCADD compared to RBC control samples. In brief, BioPAN workflow utilizes Z-score, which takes into to account both the mean and the standard deviation to assume normally distributed data of lipid subclasses and determines a reaction or pathway to be significantly modified at a *p* value <0.05 (equivalent to Z-score >1.645). The calculation of the Z-score was detailed by Gaud et al.^57^ Thus, the lipid species data set was then analyzed through BioPAN software to examine interactions between lipid subclasses. Some lipid species (95 of 267 lipid species) were not processed, as they belonged to a lipid subclass not recognized by BioPAN or were not involved in any metabolic reaction (Supplementary Table S4). BioPAN was able to sort individual lipid species into seven subclasses of lipids (PS, PE, LPE, PC, LPC, SM, and Cer). BioPAN network map of active lipid subclasses showed that the pathways corresponding to the formation of LPC from PC and LPE from PE were upregulated in MCADD patients when compared to CTRL (PC → LPC, Z-score: 4.812 and PE → LPE, Z-score: 5.023), corroborating the results described above (Figure 5 and Supplementary Table S5). Also, the conversion of PE to PC was an active reaction in MCADD RBC (Z-score: 3.217). Regarding sphingolipid metabolism, the formation of SM from Cer was also a highly active reaction (Z-score: 2.7).

**Figure 5:**
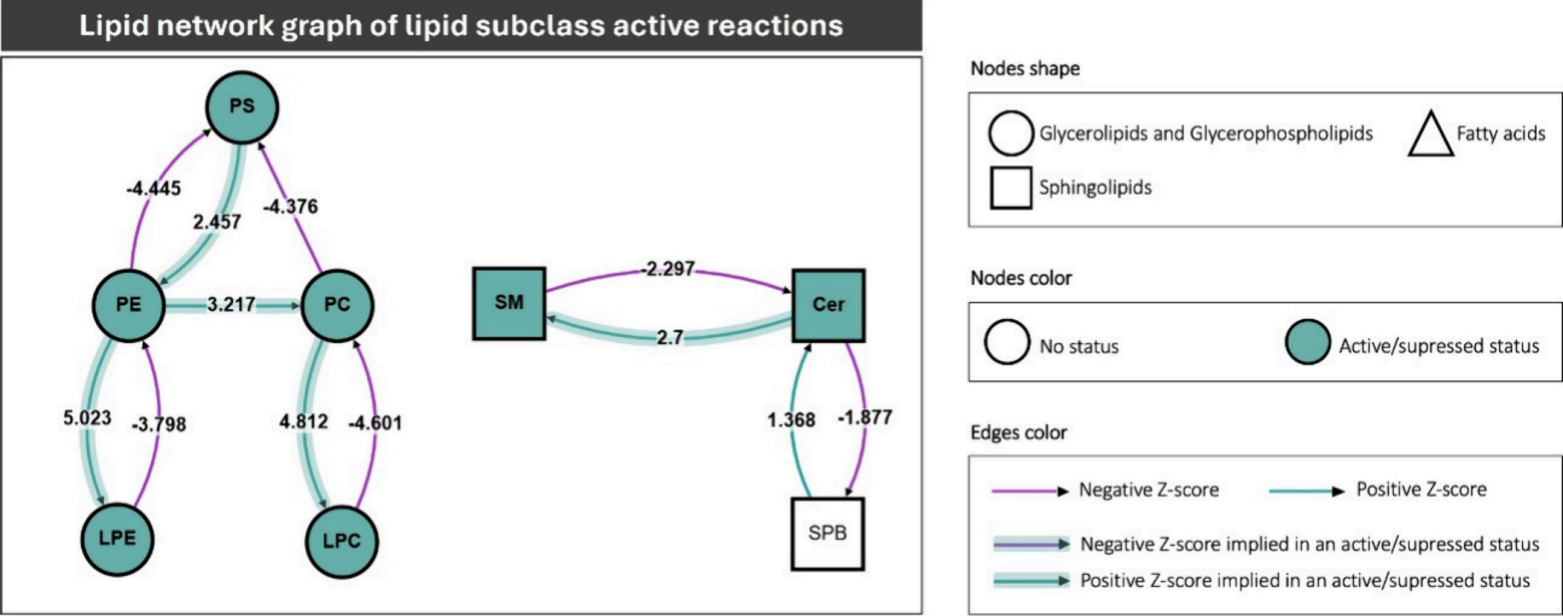
Lipid network graph generated using BioPAN software, comparing the RBC samples of medium-chain acyl-CoA dehydrogenase deficiency (MCADD) and the control (CTRL). Green nodes correspond to active lipid subclasses and green shaded arrows to active pathways. Reactions with a positive Z-score have green arrows, while negative Z-scores are colored purple. Pathways options: MCADD condition of interest, CTRL control condition, lipid type, active status, subclass level, reaction subset of lipid data, *p* value 0.05, and no paired data. Abbreviations of lipid subclasses: Cer, ceramide; LPC, lysophosphatidylcholine; LPE, lysophosphatidylethanolamine; PC, phosphatidylcholine; PE, phosphatidylcholine; PG, phosphoglycerol; PS, phosphoserine; SM, sphingomyelin; and SBP, sphingoid bases.

## 4 Discussion

Despite MCADD being generally less severe than other FAODs, it can lead to serious complications, including hepatic dysfunction and encephalopathy, if not appropriately managed through nonpharmacological intervention. Alterations in lipid homeostasis have been reported in the plasma of MCADD patients compared with control children.^13^ However, plasma is more susceptible to short-term metabolic fluctuations due to dietary intake than RBC. Indeed, RBC lipid profiling may provide a longer-term reflection of systemic lipid alterations across tissues.^24,25^ Thus, RBC may be an alternative matrix to monitor lipid plasticity in MCADD, providing more stable prognostic biomarkers for this disease.

One of the key findings of this study was the increased levels of sphingolipid species from the SM class in the RBC from MCADD patients. The observed increase in SM lipid species (Figure 2) and SM class (Figure 3) is particularly noteworthy given the crucial role of SM in cellular function and neurological health.^60,61^ SM is predominantly found in plasma membranes and lipid-rich tissue structures, such as myelin, where it contributes to membrane rigidity and stability.^60,61^ Alterations in SM metabolism have been linked to myelin destabilization^62^ and impaired synaptic signaling.^60,61^ RBC lipid membranes are known to potentially reflect systemic lipid changes, including those in the nervous system.^26,66^ While there is no direct evidence linking increased SM levels in MCADD to encephalopathy that can be observed in this disorder (primarily caused by energy deprivation) or in neurodegenerative disorders like AD,^63–65^ these findings suggest that disrupted SM homeostasis may play a role in the pathophysiology of both metabolic (MCADD) and neurodegenerative diseases. However, the specific implications of SM upregulation in MCADD require further investigation.

SM can be converted in Cer, through the action of sphingomyelinases (SMases), while Cer can also be converted back into SM by sphingomyelin synthase (SMSs).^67^ Despite the conversion of Cer into SM being one of the most active pathways in the RBC MCADD patients compared to CTRL (BioPAN analysis, Figure 5), Cer lipid species and Cer lipid class showed a remarkable increase in the RBC of MCADD patients (Figure 4). Cer accumulation has been also linked to neurodegenerative diseases^68^ and neural toxicity^69,70^ and are involved in several cellular processes, including apoptosis, oxidative stress, and neural inflammation.^71,72^ For instance, in glial cells, Cer accumulation increases reactive oxygen species (ROS) production^73^ and activates NF-kB signaling in microglia, promoting an inflammatory response. This leads to the release of proinflammatory cytokines such as TNF-α, IL-1β, and IL-6 from astrocytes, contributing to neuroinflammation and demyelination.^74,75^ Therefore, Cer upregulation is consistent with the results found for SM species and may also be a risk factor for MCADD-associated encephalopathy. However, the long-term effects of increased Cer levels in MCADD are still unknown, as the patients that were diagnosed in newborn screening and monitored afterward are still of young adult age.

Alterations in the phospholipid composition of RBC in MCADD patients were also observed, namely, increased levels of two lysophospholipid species (LPC 16:1 and LPE 18:1), alongside an increase in the LPC and LPE lipid classes (Figures 2 and 3). LPC and LPE were derived from the hydrolysis of their diacyl precursor (PC or PE) primarily via the action of phospholipases, notably phospholipase A2 (PLA2).^57^ Indeed, the conversion of PC into LPC and PE into LPE is one of the most active reactions in the BioPAN pathway analysis that was more expressed in MCADD patients (Z-score: 4.812 and 5.023, respectively, Figure 5). An increase of the LPC class is associated with enhanced inflammatory risk, partially due to the activation of NADPH oxidase,^76^ which amplifies ROS production and leads to oxidative stress. Indeed, in MCADD, oxidative stress, driven by the accumulation of medium-chain FA, is already a known issue.^8,16–20^ These changes may have implications in the inflammatory state and cellular health of MCADD patients, as lysophospholipids are bioactive lipids involved in key processes such as plasma membrane remodeling, cell growth, apoptosis, and the inflammatory cascade^57^

Considering that the LPC class is also an indication of a proinflammatory response and increase of PLA2 activity,^57^ the PC/LPC ratio was assessed. The decreased PC/LPC ratio observed in RBC of MCADD patients together with the BioPAN analysis (PC → LPC, Z-score: 4.812) suggests an enhanced conversion of PC into LPC. This ratio mirrors the downregulation observed in the polyunsaturated PC species observed in the RBCs of MCADD patients and is also in agreement with the pattern observed for the decrease in PC class. Thus, compared to the RBC of CTRL individuals, MCADD patients exhibited a decrease in lipid species belonging to the PC and PE classes. These diminished PC and PE lipid species, including both diacyl- and ether-linked forms, were predominantly characterized by the presence of esterified polyunsaturated FA (PUFA). Phospholipids enriched with PUFA, such as arachidonic acid and docosahexaenoic acid, play a dual role as precursors for bioactive lipid mediators and as critical antioxidants, scavenging ROS to mitigate oxidative damage.^77^ Furthermore, the reduction of ether-linked PC and PE in MCADD RBCs, particularly of plasmalogen species, well known for their role in antioxidant defenses,^78^ may also contribute to the redox imbalance.^8,16–20^ The lower abundance of plasmalogen species in the MCADD RBC might serve as a biomarker for systemic oxidative stress.

Although both lipid classes, PC and PE, showed a decrease in the RBCs of MCADD patients, an increase in the PC/PE ratio was observed. This can be explained by the increased activity of the Kennedy pathway (which converts PE to PC), through the action of PE *N*-methyltransferase (PEMT). Also, the higher PC/PE ratio in MCADD patients suggests a shift toward increased membrane fluidity.^79,80^ An increase in the PC/PE ratio was previously associated with the deregulation in mitochondrial biogenesis, affecting energy production.^80,81^ In fact, mitochondrial dysfunction is a hallmark of MCADD.^82^ Furthermore, the increased (PC + SM)/(PE + PS) ratio in MCADD patients indicates a potential disruption in membrane asymmetry.^31,58^ PC and SM are primarily localized in the outer leaflet of the RBC membrane, while PE and PS are typically concentrated in the inner leaflet. An imbalance in this distribution suggests a disturbance in the lipid bilayer’s structural organization, which could impact membrane stability and functionality.^31,58^ All these alterations may impair RBC functions, as the fluidity and deformability of the RBC membrane must be maintained to allow it to pass through small capillaries for oxygen delivery^83,84^

It is worth noting that we observed distinct lipid changes in RBCs that differ from those previously reported for plasma.^13^ In MCADD patients, RBCs exhibited an increase in sphingolipid subclass, including SM, Cer, and HexCer, compared to the CTRL group. Conversely, a previous study with plasma of MCADD patients reported decreased SM and Cer. Nevertheless, in both plasma and RBCs from MCADD patients, a decrease in PC (diacyl and ether-linked) and PE (diacyl) species was noticed.^13^ A previous study comparing plasma and RBCs pointed that sphingolipids are more abundant and show better interday reproducibility in RBCs.^56^ While the use of RBC remains less established in the clinical lipidomics field compared to plasma, our findings suggest that differences at lipid classes, namely, in sphingolipids, may favor the use of RBC.

Based on the lipidomics analysis, this study reveals alterations in the RBC lipids of children with MCADD. However, it is important to recognize a few limitations. MCADD is a rare condition, and our study population consists of children, making sample collection for research purposes particularly challenging. A number of 73 RBC samples (35 from MCADD and 38 from CTRL) were analyzed, within the same range as other MCADD studies.^11–13^ Larger cohort studies are needed to confirm whether the lipidomic alterations observed are specific to MCADD. In addition, longitudinal analyses will be crucial to monitor changes in the lipid profile over time and to investigate their relationship with the development of possible comorbidities. Understanding lipidomic changes in MCADD may ultimately aid in monitoring disease progression and identifying late-onset complications.

## 5 Conclusions

Our results revealed that MCADD significantly alters lipid metabolism, leading to distinct changes in the RBC membrane lipid profile. These alterations include an increase in sphingolipids (SM and Cer) and lysophospholipids (LPC and LPE), alongside a decrease in PUFA-containing and ether-linked phospholipids (PC and PE). The elevated sphingolipids and lysophospholipids suggest potential association with oxidative stress, inflammation, and neurological complications. Simultaneously, the depletion of PUFA-containing phospholipids and plasmalogens compromises the antioxidant defenses possibly exacerbating oxidative stress.

These findings highlight the importance of RBC lipidomic analysis in understanding the systemic and long-term metabolic impacts of MCADD by providing valuable awareness of the metabolic changes in the lipid profile associated with MCADD and their potential link to comorbidities. This knowledge is especially relevant given the emerging evidence linking early life lipidomic shifts to adult-onset comorbidities. The study of lipidomic alterations in children with MCADD may provide important insights into the pathogenesis and monitoring of disease progression, with the aim of early detection and eventually prevention of long-term complications.

## Supplementary Material





## Data Availability

The data sets supporting this article have been uploaded as part of the supporting material. Data for this paper, including raw data files are available at Science Data Bank, 2025 [2025-04-22] at https://www.scidb.cn/en/s/meU7Rv. CSTR: 31253.11.sciencedb.23952. Data DOI: 10.57760/sciencedb.23952. https://www.scidb.cn/en/s/MnInAz.https://www.scidb.cn/en/anonymous/bWVVN1J2

## Notes

Variables ordered by contribution (%) to dimension (Dim) 1 of the principal component analysis (PCA) LC-MS data set (Figure S1); variables ordered by contribution (%) to dimension (Dim) 2 of the principal component analysis (PCA) of LC-MS data set (Figure S2) (PDF)
Demographic and clinical data collected for each patient with medium-chain acyl-CoA dehydrogenase deficiency (MCADD) (Table S1); lipids identified by C18-LC-MS and MS/MS of total lipid extracts obtained from RBC samples collected from MCADD patients and controls (mass error <5 ppm) (Table S2); 240 lipid species showing significant variations between MCADD and control (CTRL) groups (Table S3); lipid species that were processed/unprocessed by the BioPAN software, either because they belong to lipid subclasses not recognized by BioPAN or because they are not involved in any metabolic reactions (Table S4); BioPAN results by comparing the samples from MCADD RBC with the CTRL RBC (Table S5) (XLSX)
