# Supplementary material for: Lipidomic Profiling of Red Blood Cells in the Mitochondrial Fatty Acid β‑oxidation Disorder MCADD Reveals Phospholipid and Sphingolipid Dysregulation
Source: J Proteome Res. 2025 Jul 24;24(9):4631–42. doi: 10.1021/acs.jproteome.5c00308 (PMC12818831; doi:10.1021/acs.jproteome.5c00308)
Supplement: Supplementary file 1 [file pr5c00308_si_001.pdf]

Supplementary Information

Lipidomic Profiling of red blood cells in the mitochondrial fatty acid β-oxidation disorder MCADD Reveals Phospholipid and Sphingolipid Dysregulation

Inês M. S. Guerra[a,b], Helena B. Ferreira[a], Luísa Diogo[c,d], Sónia Moreira[c,d], Stefano Bonciarelli[e], Laura Goracci[f], Tânia Melo[a,b], Pedro Domingues[a], M. Rosário Domingues[a,b], Ana S. P. Moreira[a,b,*]

[a] Mass Spectrometry Center, LAQV-REQUIMTE, Department of Chemistry, University of Aveiro, Campus Universitário de Santiago, 3810-193 Aveiro, Portugal

[b] CESAM - Centre for Environmental and Marine Studies, Department of Chemistry, University of Aveiro, Campus Universitário de Santiago, 3810-193 Aveiro, Portugal

[c] Centro de Referência de Doenças Hereditárias do Metabolismo, Unidade Local de Saúde de Coimbra, MetabERN (Portugal)

[d] Serviço de Bioquímica, Faculdade de Medicina da Universidade de Coimbra (Portugal)

[e] Molecular Discovery Ltd., HertfordshireWD6 4PJ, U.K

[f] Department of Chemistry, Biology and Biotechnology, University of Perugia (Perugia, Italy)

***Corresponding author:** Ana S. P. Moreira

E-mail address: ana.moreira@ua.pt (Ana Moreira).

Table of contents:

Figures

Supplementary Figure S1. Variables ordered by contribution (%) to dimension (Dim) 1 of the principal component analysis (PCA) LC-MS dataset.

Supplementary Figure S2. Variables ordered by contribution (%) to dimension (Dim) 2 of the principal component analysis (PCA) of LC-MS dataset.

Tables (uploaded separately in xlsx file)

Supplementary Table S1. Demographic and clinical data collected for each patient with medium-chain acyl-CoA dehydrogenase deficiency (MCADD).

Supplementary Table S2. Lipids identified by C18-LC-MS and MS/MS of total lipid extracts obtained from RBC samples collected from MCADD patients and controls (mass error < 5 ppm).

Supplementary Table S3. The 240 lipid species showing significant variations between MCADD and control (CTRL) groups.

Supplementary Table S4. Lipid species that were processed/unprocessed by the BioPAN software, either because they belong to lipid subclasses not recognized by BioPAN or because they are not involved in any metabolic reactions.

Supplementary Table S5. BioPAN results by comparing the samples from MCADD RBC with the CTRL RBC.

Highlights:

- MCADD significantly alter the lipid profile of red blood cells (RBC).
- MCADD showed up-regulation in SM, Cer, LPC and LPE, linked to oxidative stress and inflammation.
- Down-regulation of PUFA-containing and ether-linked phospholipids may compromise the antioxidant defences.
- Increased PC/PE and (PC+SM)/(PE+PS) ratios suggest changes in MCADD RBC membranes properties.
- RBC lipidomics may be a useful tool to monitor MCADD progression.

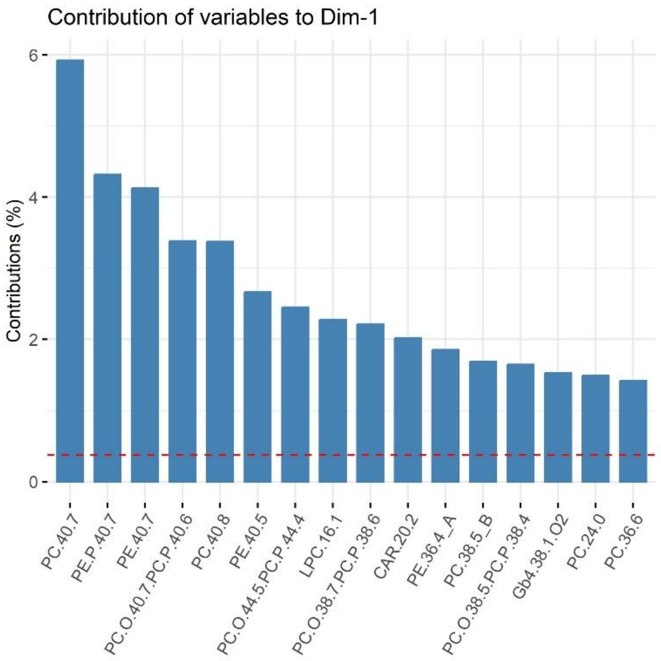

Supplementary Figure S1. Variables ordered by contribution (%) to dimension Dim 1 of the principal component analysis (PCA) of LC-MS dataset.

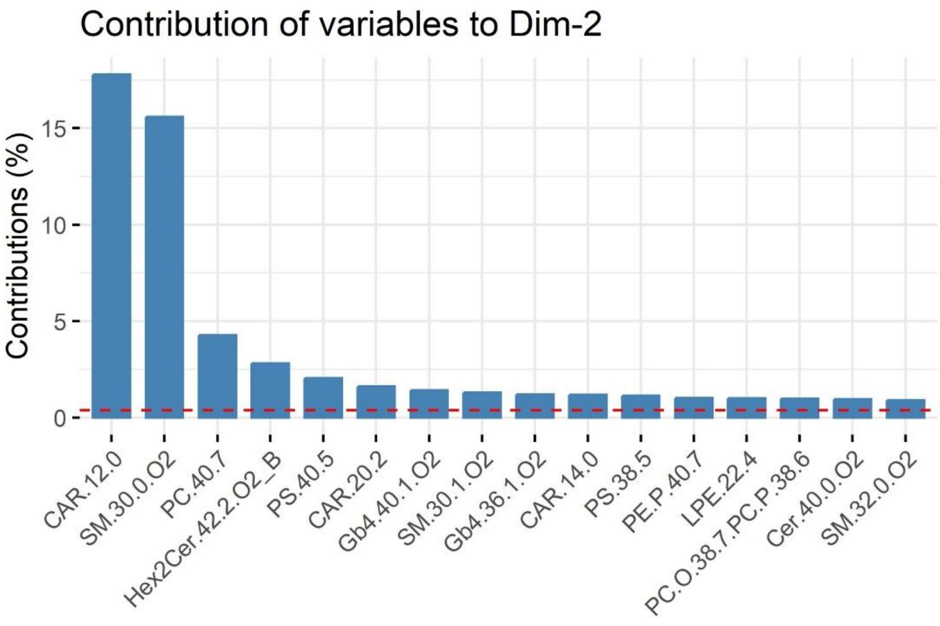

Supplementary Figure S2. Variables ordered by contribution (%) to dimension Dim2 of the principal component analysis (PCA) of LC-MS dataset
